# Designer Self-Assembling Peptide Nanofiber Scaffolds Containing Link Protein N-Terminal Peptide Induce Chondrogenesis of Rabbit Bone Marrow Stem Cells

**DOI:** 10.1155/2014/421954

**Published:** 2014-08-26

**Authors:** Baichuan Wang, Caixia Sun, Zengwu Shao, Shuhua Yang, Biao Che, Qiang Wu, Jianxiang Liu

**Affiliations:** ^1^Department of Orthopedics, Union Hospital, Tongji Medical College, Huazhong University of Science and Technology, Wuhan 430022, China; ^2^Department of Gynecology, General Hospital of the Yangtze River Shipping, Wuhan 430022, China; ^3^Department of Orthopedics, General Hospital of the Yangtze River Shipping, Wuhan 430022, China

## Abstract

Designer self-assembling peptide nanofiber hydrogel scaffolds have been considered as promising biomaterials for tissue engineering because of their excellent biocompatibility and biofunctionality. Our previous studies have shown that a novel designer functionalized self-assembling peptide nanofiber hydrogel scaffold (RLN/RADA16, LN-NS) containing N-terminal peptide sequence of link protein (link N) can promote nucleus pulposus cells (NPCs) adhesion and three-dimensional (3D) migration and stimulate biosynthesis of type II collagen and aggrecan by NPCs *in vitro*. The present study has extended these investigations to determine the effects of this functionalized LN-NS on bone marrow stem cells (BMSCs), a potential cell source for NP regeneration. Although the functionalized LN-NS cannot promote BMSCs proliferation, it significantly promotes BMSCs adhesion compared with that of the pure RADA16 hydrogel scaffold. Moreover, the functionalized LN-NS remarkably stimulates biosynthesis and deposition of type II collagen and aggrecan. These data demonstrate that the functionalized peptide nanofiber hydrogel scaffold containing link N peptide as a potential matrix substrate will be very useful in the NP tissue regeneration.

## 1. Introduction 

Intervertebral disc (IVD) degeneration is major cause of chronic low back pain [1]. Epidemiological studies showed that 54%–80% of people experienced chronic low back pain during their lifetimes and the annual prevalence of chronic low back pain was reported as 15%–45% [2, 3]. Repair of degenerated IVDs remains a challenge, despite the development of surgical interventions such as nucleotomy [4] and spinal fusion [5], which are used with the goal of relieving symptomatic pain rather than curing the diseases. With the development of cellular biology and new biomaterials, NP tissue engineering may offer a new therapeutic strategy for IVDs degeneration.

Cell population changes in degenerating IVDs have been implicated in the pathogenesis of IVD degeneration. Cells of degenerating discs—whether resulting from their declining numbers, change in phenotype, or replacement by less effective cells—appear to be unable on their own to produce functional ECM [6–8]. Therefore, a critical factor for NP tissue regeneration is “more effective cells.” NPCs from mature tissues can be difficult due to problems with limited harvest sites and expansion capacity. Recently, bone marrow stem cells (BMSCs) have been used as a cell source for NP tissue engineering strategies aimed at reconstructing regenerated IVDs. BMSCs can differentiate into chondrocyte phenotype cells and produce a cartilage-like matrix when encapsulated in a variety of three-dimensional (3D) scaffolds [9–11]. Moreover, the synergistic effect between nucleus pulposus cells (NPCs) and BMSCs promoted BMSCs differentiation towards NPCs-like phenotype and increased the functional ECM synthesis rate [8].

In addition to suitable seed cells, excellent bioscaffold is another critical factor for tissue engineering. An ideal NP tissue engineering scaffold should be biocompatible and provide appropriate cell microenvironment and differentiation cues. Self-assembling peptides are a new class of molecules which have the capacity to form stable nanofiber hydrogels [12–16]. These self-assembling peptide hydrogel scaffolds have been used for cell 3D culture and tissue repair and exhibited excellent biocompatibility [12–20]. Moreover, self-assembling peptides can be functionalized with adhesion and signaling motifs to improve cell adhesion, proliferation, and 3D migration and induce cell differentiation [13, 14, 16]. RADA16 hydrogel, a typical peptide self-assembling hydrogel, has been used for cartilage tissue regeneration [12, 16, 19, 20]. Although RADA16 hydrogel scaffold can maintain chondrocyte phenotype [19] and stimulate chondrogenesis of BMSCs [20], it does not contain any active motif [21]. These exciting results may be attributed to the suitable cell microenvironment provided by RADA16 scaffold. In order to provide an improved chondrogenic microenvironment, we obtained functionalized LN-NS formed by mixing designer self-assembling peptide RLN containing the N-terminal peptide sequence of link protein (link N) and RADA16, and our previous studies showed that the functionalized LN-NS can maintain NPCs (also called chondrocyte-like) phenotype and promote biosynthesis of aggrecan and type II collagen [12, 16]. Moreover, a recent study also showed that link N peptide can act as “BMP-like” to influence cell biobehaviors [22]. These data indicate the functionalized LN-NS may have a significant effect on the stem/progenitor cells such as BMSCs.

In this study, we have extended our investigations to observe the effect of the functionalized LN-NS on BMSCs. We tested the biocompatibility of the functionalized LN-NS and assessed the influence of the functionalized LN-NS on cell adhesion. The biosynthesis of ECM was also evaluated by quantitative real-time PCR and immunofluorescence. Our findings demonstrate that the functionalized LN-NS can significantly promote cell adhesion and upregulate synthesis of proteins, including aggrecan and type II collagen. This study may provide a better understanding of the novel functionalized LN-NS.

## 2. Materials and Methods

### 2.1. Isolation and Culture of BMSCs

Bone marrow was extracted from the femurs of 3 Japanese white rabbits (3 weeks old, Hubei Research Center of Laboratory Animal, China) and BMSCs were isolated as described previously [23]. Nucleated cells were isolated with a density gradient (Boster, China) and washed twice with standard cell culture medium, Dulbecco's modified eagle medium: nutrient mixture F-12 (DMEM-F12) (Hyclone). These cells were cultured in maintenance medium containing DMEM-F12, 10% fetal bovine serum (FBS) (Sigma), and penicillin streptomycin amphotericin (100 U/mL penicillin, 100 *μ*g/mL, and 250 ng/mL) and incubated at 37°C in a humidified atmosphere chamber with 5% CO_2_. After 48 h, nonadherent cells were discarded, and the remaining adherent cells were thoroughly washed twice with phosphate buffered saline (PBS) (Boster) and cultured for a further 3–7 days. Upon achieving 80–90% confluence, the cells were treated with 0.25% trypsin for 3–5 min at 37°C, washed 2 times with culture medium and replanted in another culture flask.

### 2.2. Hydrogel Preparation and Cell Seeding

The designer peptide RLN with sequence (AcN-(RADA)_4_-GGRLNSDNYTLDHDRAIH-COHN_2_) and pure peptide RADA16 with sequence (AcN-(RADA)_4_-COHN_2_) were synthesized and purified by Shanghai Jier biotechnology Co., China. The functionalized LN-NS and RADA16 hydrogels were prepared according to our previously reported methods [16]. RADA16 hydrogel (1%) was prepared by dissolving peptide powder with 10% sucrose solution. For preparation of the functionalized LN-NS hydrogel (1%), 5 mg of RLN peptide powder was dissolved with 500 *μ*L of 10% sucrose solution and mixed with 500 *μ*L of RADA16 solution. Before cell encapsulation, these peptide hydrogels were treated with DMEM-F12 solution, every 4 h within 12 h to equilibrate the growth environment to physiological pH. BMSCs from the third passage were harvested with 0.25% trypsin. For cell adhesion tests, BMSCs were seeded on the top-surface of each hydrogel at a final concentration of 1 × 10^6^ cells/mL. In the case of cytotoxicity, cell proliferation and differentiation tests, BMSCs were encapsulated in 1% functionalized LN-NS or 1% RADA16 hydrogel at a concentration of 5 × 10^6^ cells/mL. Cells/hydrogels suspension was slowly dropped onto the bottom surface of each cell culture well/insert (well diameter: 4.5 mm; insert diameter: 6.5 mm, pore sizes: 8 *μ*m) and allowed to form a layer ~1.0 mm thick. DMEM-F12 solution was gently added to enhance self-assembly, and then these wells/inserts were incubated at 37°C for 30 min to complete the gelation. Subsequently, these cells/hydrogels systems were cultured in maintenance medium or differentiation medium. The differentiation medium was serum-free medium, containing DMEM (high glucose), 1% ITS+1 (insulin, transferring and selenium, Sigma), bovine serum albumin 1.25 mg/mL, pyruvate 1 mM, linoleic acid 5.35 mg/mL, and ascorbate 2-phosphate 50 mg/mL. In this study, all experiments were performed at least three times.

### 2.3. Microstructure of Peptide Scaffolds and Rheological Analysis

The microstructure of peptide scaffolds was observed by an atomic force microscopy (AFM, DI Nanoscope IV, Veeco probe Inc., CA). After sonication for 30 min, each peptide solution (RLN, RADA16, and RLN/RADA16 mixture) was diluted to a working concentration of 0.01%. 5 *μ*L of peptide sample was immediately deposited onto mica for 25–30 seconds and then washed two times with 100 *μ*L of deionized water. These peptide samples on the mica surface were stationary incubated at room temperature for 3-4 hours, and the microstructure of peptide samples was observed using AFM.

Rheological data were obtained by an AR 2000 rheometer (TA Instruments, New Castle, DE). After sonication for 30 min, 50 *μ*L of peptide solution (1%, RADA16, RLN, RLN/RADA16 mixture) was placed on the plate of the rheometer and a gap of 250 *μ*m was established. These peptide solutions were allowed to stand for 30 min for gelating on the plate. Strain sweep experiments (0–10%) at oscillatory frequency of 10 Hz were performed to determine the linear viscoelastic regime. In this study, the storage modulus (*G*′) and loss modulus (*G*′′) were measured at 25°C, and the parameters used for the frequency sweep tests were strain 1% and frequency range 0–100 rad/sec.

### 2.4. Cytotoxicity Assay and Cell Morphology Observation

BMSCs suspension was mixed with peptide solution (RADA16 or RLN/RADA16 mixture) and dropped onto the cell culture inserts. A LIVE/DEAD cell viability kit (calcein-am/PI) was used to evaluate the number of live cells and dead cells encapsulated in these peptide hydrogels. After 3 and 7 days of culture, calcein-am (5 *μ*g/mL) and PI (5 *μ*g/mL) were added into cells/hydrogels medium and incubated for 30 min at room temperature in the dark. Then cells/hydrogels were gently rinsed 3 times with PBS solution and observed under a fluorescence microscope (TE2000-U, NIKON, Japan). The number of live cells and total cells were counted in five randomly selected nonoverlapping fields, and cell morphology was observed in a random field.

### 2.5. Cell Adhesion Assay

In the case of cell adhesion tests, cell culture wells (well diameter: 4.5 mm) were coated with functionalized LN-NS and RADA16 hydrogel and then treated with DMEM-F12 solution to enhance peptide self-assembly. 100 *μ*L of BMSCs suspension was added to the top-surface of peptide hydrogels and incubated for 1, 2, 3, 4, 5, and 6 h. In each time point, cells/hydrogels were rinsed gently 3 times using PBS solution (30 s/rinse) to remove unattached cells, and cell counting kit-8 (CCK-8) (Dojindo, Japan) reagent was added to evaluate the number of the remaining attached cells. Briefly, 10 *μ*L of CCK-8 reagent was added into 100 *μ*L of cells/hydrogels medium and incubated for 2 h at 37°C in the dark. Absorbance indirectly reflecting the number of attached cells was measured at 450 nm using an enzyme-labeled instrument (Elx800, Bio-Tek, USA).

### 2.6. Cell Proliferation Assay

A previous study showed that peptide in digested samples could interfere with DNA content detection [24]. Therefore, we used a viable cell counting kit (CCK-8 kit) to measure viable cells encapsulated in the peptide hydrogels, which is similar to the method described by Kisiday et al. [15]. To eliminate the influence of peptide hydrogels on absorbance, eight wells with acellular hydrogels (RADA16 or functionalized LN-NS) were set to get average absorbance of peptide hydrogels. On days 2, 4, and 6, cells/hydrogels were punched and incubated with CCK-8 reagent. CCK-8 reagent was used as the procedure described in cell adhesion tests. The absorbance of medium samples at 450 nm was measured after 2 h.

### 2.7. Quantitation Assay of Chondrogenic Genes (RT-PCR)

After 7 and 14 days of culture, cells/hydrogels were disrupted mechanically after adding 500 *μ*L of DMEM-F12 solution and then centrifuged (200 ×g). The harvested cell pellets were rinsed twice with PBS solution. Total RNA was extracted from cultured cells using TRIzol Reagent (Invitrogen, USA), and absorbance measured at 280 nm was used to determine the diluted RNA concentration. 1 *μ*g of total RNA was reversely transcribed using ReverTra Ace reverse transcriptase reaction system (Toyobo, Japan). The rabbit specific primers, type II collagen (*coll 2*) and proteoglycan (*agg*), were designed according to our previous published primer sequences [16]. The amount of PCR products was estimated by measuring the intensity of the fluorescence of SYBR green embedded into the double stranded DNA. The mRNA expression levels of target genes were calibrated using housekeeping gene glyceraldehyde-3-phosphate dehydrogenase (GAPDH) expression levels as an internal control.

### 2.8. Collagen Immunofluorescence Assay

To observe visually the effect of the functionalized LN-NS on the biosynthesis of ECM, BMSCs were seeded on the surface of peptide hydrogels for 14 days. On day 14, cells/hydrogels were gently rinsed 3 times with PBS solution and treated with 0.2% Triton X-100 (Sigma) for 5 min. After blocking heterogenetic antigens, lineage-specific primary antibody, anti-collagen II (1 : 50, Bios), was added into the cells/hydrogels and incubated overnight at 4°C. Cells/hydrogels were then rinsed 3 times with PBS solution and incubated for 1 h at 37°C in the dark with FITC-conjugated secondary goat anti-rabbit IgG (1 : 250, Boster). Cell nucleuses were stained with Hoechst 33342 (Sigma) for 30 min in the dark. Cells/hydrogels samples were observed by a laser confocal microscopy (LCM, FV500, Olympus, Japan).

### 2.9. Statistical Analysis

All data were expressed as mean ± standard deviation (M ± SD). Differences between LN-NS and RADA16 hydrogel were analyzed by the paired Student's *t*-test with *P* < 0.05 reported as statistical significance.

## 3. Results

### 3.1. Self-Assembly of Peptides and Microstructure of Hydrogel Scaffolds

**Figure 1:**
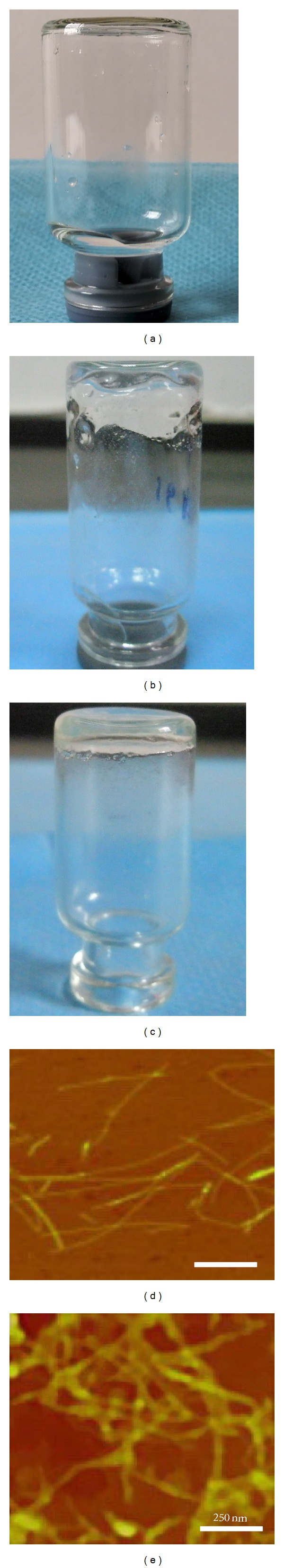
Self-assembly of peptide and microstructure of peptide scaffolds. (a) RLN peptide solution. (b) A self-supporting hydrogel was made by the mixture of RLN and RADA16, and self-assembly of peptide was enhanced by CaCl_2_ (0.1 mol/L) solution (c). The nanofibers were observed in RADA16 (d) and LN-NS (e). There was an increase of the diameter of nanofibers of LN-NS (31.9 ± 3.8 nm) compared with that of RADA16 (13.5 ± 1.8 nm).

After dissolving peptide powder in 10% sterile sucrose, RADA16 formed a transparent viscous hydrogel, while RLN remained as a nonviscous solution. However, a hydrogel was formed when RLN was mixed with RADA16 at a ratio 1 : 1, and the gel strength of the hydrogel could be enhanced by CaCl_2_ (0.1 mol/L) solution (Figures 1(a)–1(c)).

We studied the microstructure of 1% peptide solution of RADA16, RLN, and RLN mixed with RADA16 at a ratio 1 : 1 (LN-NS) using AFM. There was no nanofiber formation in 1% of RLN, but the nanofibers were observed in 1% of RADA16 and LN-NS. The diameter of the nanofiber self-assembled by LN-NS was 31.9 ± 3.8 nm, while the diameter of nanofibers of RADA16 was 13.5 ± 1.8 nm. The length of these nanofibers could be ranging from several hundred nanometers to a few microns (Figures 1(d) and 1(e)).

### 3.2. Rheological Analysis

**Figure 2:**
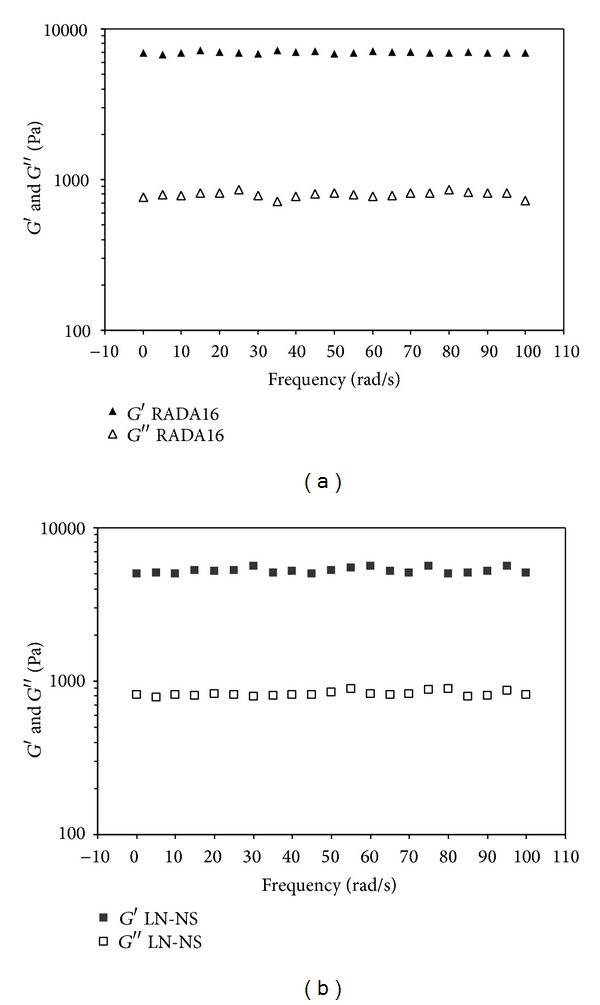
Rheological analysis of peptide hydrogels. (a) RADA16 hydrogel, (b) LN-NS. Storage modulus (*G*′) and loss modulus (*G*′′) of RADA16 and LN-NS as a function of frequency (measurements were performed at 1% stain and 25°C). The value of *G*′ was significantly larger than that of *G*′′ both in RADA16 and LN-NS.

In the strain sweep experiments, only RADA16 and LN-NS had identifiable linear viscoelastic regions, whereas the plain RLN did not. This was probably because the plain RLN was too fragile and could not withstand small levels of shear stress. The storage modulus (*G*′) and loss modulus (*G*′′) responded to the elasticity and viscosity of materials, respectively. As it shows in Figure 2, the frequency sweep results indicated that the value of the storage modulus (*G*′) was larger than that of the loss modulus (*G*′′) both in RADA16 and LN-NS, and both *G*′ and *G*′′ were independent of frequency in the whole experiments.

### 3.3. Cytotoxicity Assay and Cell Morphology Observation

**Figure 3:**
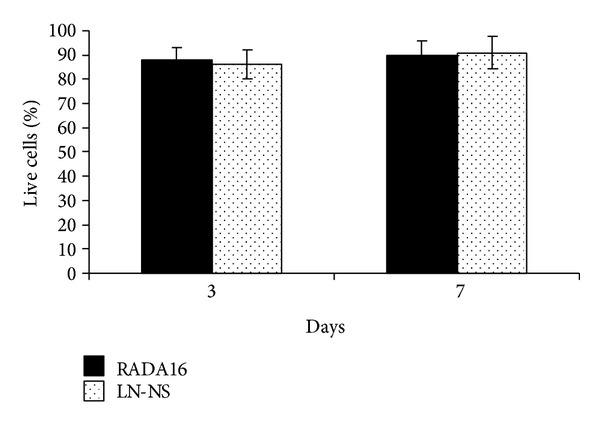
Quantification assay of cell survival rates. There was no significant difference in cell survival rates between functionalized LN-NS and pure RADA16 hydrogel after 3 and 7 days of culture (*P* > 0.05).

**Figure 4:**
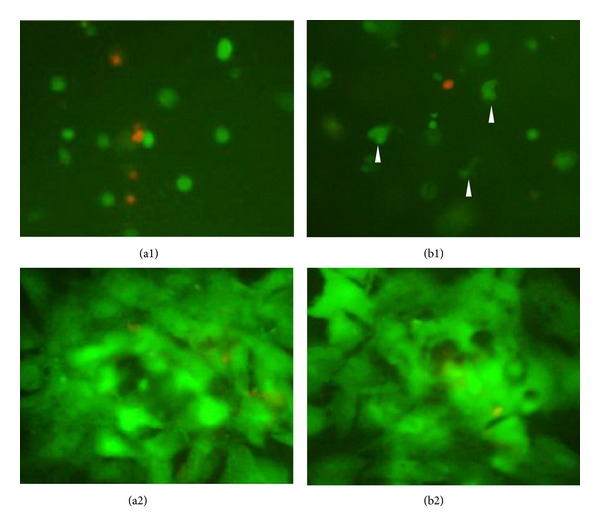
Cell viability and morphology of BMSCs encapsulated in ((a1) ×200; (a2) ×400) RADA16 hydrogel and ((b1) ×200; (b2) ×400) LN-NS hydrogel after 3 days and 7 days of culture. Stained with calcein-am/PI, the live cells had green fluorescence and the dead and dying cells showed red fluorescence. Some cells encapsulated in LN-NS showed triangular or polygonal morphology at day 3 (arrow), but most cells in the two peptide hydrogels showed similar cell morphology after 7 days of culture.

To analyze the effect of the functionalized LN-NS on cell viability, we used a LIVE/DEAD cell viability kit (calcein-am/PI) to observe live cells and dead cells encapsulated in the peptide hydrogel. The pure RADA16 hydrogel was used as control. The live cells produce intense uniform green fluorescence because of calcein-am dye in response to intracellular ubiquitous esterase activity, and the dead and dying cells produce bright red fluorescence as a result of the binding of PI dye and nucleic acids. As it shows in Figures 3 and 4, the functionalized LN-NS possessed similar cells-scaffold biocompatibility when compared with the pure RADA16 hydrogel after 3 and 7 days of culture (*P* > 0.05). Interestingly, some cells encapsulated in the functionalized LN-NS showed triangular or polygonal morphology at day 3, but most cells in the two peptide hydrogels showed similar cell morphology after 7 days of culture, which might be a feature of cell-cell communication (Figure 4).

### 3.4. Effect of Functionalized LN-NS Hydrogel on Cell Adhesion

**Figure 5:**
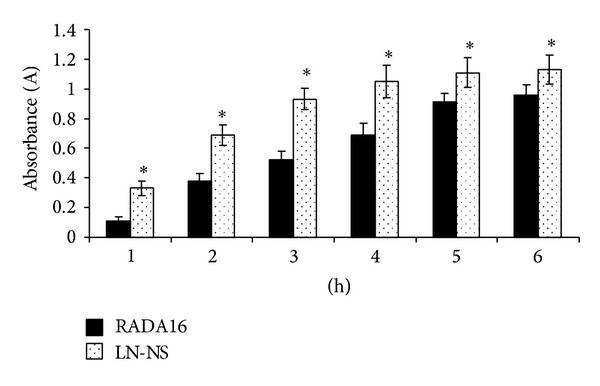
Effects of peptide hydrogels on cell adhesion. Significant cell adhesion on the functionalized LN-NS was observed just at 1 h, which was faster than the pure RADA16 hydrogel. In the 6 hours of culture, the number of cells attached on the functionalized LN-NS was significantly higher when compared with that of pure RADA16 hydrogel (_ _*P < 0.05).

Cells-scaffold adhesion is a critical factor for tissue engineering biomaterials [25]. RADA16 hydrogel was widely used as a substrate for culturing a variety of cells and promoted cell adhesion [12, 16, 26]. In our experiments, we used the pure RADA16 hydrogel as control to evaluate the effect of the functionalized LN-NS on cell adhesion. BMSCs were seeded onto the top-surface of these peptide hydrogels for 1, 2, 3, 4, 5, and 6 h, respectively. As expected, the functionalized LN-NS containing link N motif significantly promoted BMSCs adhesion in comparison to the pure RADA16 hydrogel (*P* < 0.05). Although the number of cells attached on the two peptide hydrogels continued to increase with time during the 6 hours of culture, the functionalized LN-NS significantly increased cell adhesion just at 1 h (Figure 5).

### 3.5. Effect of Functionalized LN-NS Hydrogel on Cell Proliferation

**Figure 6:**
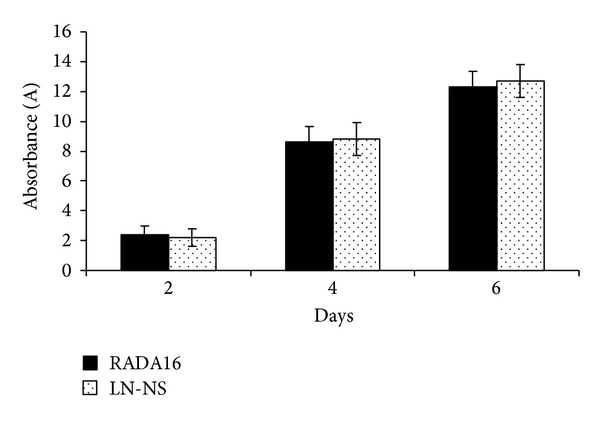
Effects of peptide hydrogels on cell proliferation. The similar cell growth was observed in the functionalized LN-NS and pure RADA16 hydrogel during 6 days of culture (*P* > 0.05).

We evaluated cell growth in peptide hydrogels by growing BMSCs in the RADA16 and functionalized LN-NS hydrogel for 2, 4, and 6 days. The number of cells was evaluated by a CCK-8 cell counting kit, and the absorbance indirectly reflecting the number of cells was shown in Figure 6. Cell proliferation was observed in the two peptide hydrogels, but there was no significant difference between functionalized LN-NS and pure RADA16 hydrogel (*P* > 0.05). The similar cell growth in the two peptide hydrogels may be as a result of rapid proliferation of BMSCs during 6 days of culture or indicates that the link N peptide incorporated in the functionalized LN-NS may have no effect on promoting BMSCs proliferation.

### 3.6. Effect of Functionalized LN-NS on Expression of Chondrotype-Related Genes

**Figure 7:**
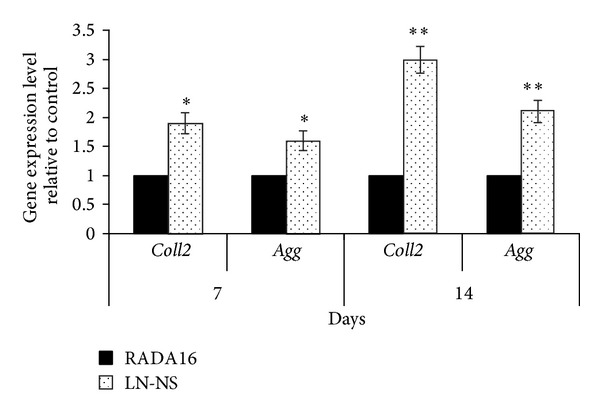
Quantitative analysis of chondrocyte-related genes expression in cells encapsulated in functionalized LN-NS and RADA16 hydrogel at 7 and 14 days. In comparison to pure RADA16 hydrogel, the functionalized LN-NS significantly promotes the expression of chondrocyte-related genes (_ _*P < 0.05, _ _**P < 0.01).

Gene expression levels were analyzed by SYRB green quantitative RT-PCR. In our experiments, we assayed two chondrotype specific genes: type II collagen (*coll 2*) and proteoglycan (*agg*). Although the expression levels of* coll 2* and* agg* mRNA continued to increase in these peptide hydrogels from day 0 to day 14, the functionalized LN-NS significantly increased the expression of* coll 2* and* agg* when compared with the pure RADA16 hydrogel. As it shows in Figure 7,* Coll2* mRNA in BMSCs encapsulated in the functionalized LN-NS increased 90% when compared with the pure RADA16 hydrogel on day 7, and there was a 200% increase on day 14. Consistent with the expression of* coll2* mRNA,* agg* mRNA was also upregulated.* Agg* mRNA in BMSCs encapsulated in the functionalized LN-NS was 60% higher than in the RADA16 hydrogel on day 7, and there was a 110% increase on day 14.

### 3.7. Effect of Functionalized LN-NS on Biosynthesis of ECM

**Figure 8:**
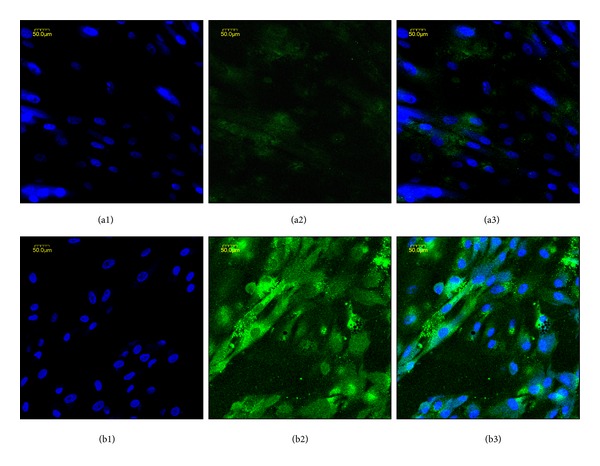
Deposition of type II collagen in cells cultured on the RADA16 hydrogel (a1, a2, and a3) and functionalized LN-NS (b1, b2, and b3) at 14 days. Stained with Hoechst dye (a1 and b1) and FITC-conjugated anti-collagen II antibody (a2, b2), the cell nuclei had blue fluorescence, and type II collagen had green fluorescence. Immunofluorescence showed these cells on the functionalized LN-NS exhibited a higher differentiated level when compared with the pure RADA16 hydrogel.

The protein production of* coll 2* and* agg* is closely related to the cellular corresponding genes expression levels [27, 28], but we still stained type II collagen in culture cells in order to get further confirmation. To observe visually the biosynthesis of type II collagen, cells were seeded on the peptide hydrogels for 14 days and stained with immunofluorescence. Previous studies showed that the pure RADA16 hydrogel can stimulate chondrogenesis of rabbit BMSCs [20]. However, cells on the functionalized LN-NS exhibited a higher expression level of type II collagen when compared with the pure RADA16 hydrogel as it is shown in Figure 8. These data of RT-PCR and immunofluorescence suggest that the functionalized LN-NS containing link N peptide can provide improved chondrogenic differentiation microenvironment for BMSCs, and these exiting results may be attributed to the bioactive link N peptide.

## 4. Discussion

Normal NP tissue is in a state of dynamic equilibrium, in which the anabolic and catabolic processes of NPCs within ECM are regulated by a range of growth factors. In the degenerated NP tissues, this balance is disturbed, which results in the loss of matrix components and intervertebral disc height and subsequent chronic low back pain. Therefore, research is now focusing on promoting regeneration of degenerating NPs by stimulating production of ECM. Previous studies showed that transforming growth factor-*β*1 (TGF-*β*1), platelet-rich plasma (PRP), and BMP can stimulate the production of cartilage or NPs matrix* in vitro* and* in vivo* [29–31]. More recently, there have been reports showing that a number of peptide fragments generated from matrix degradation have a major influence on biosynthesis or degradation of ECM [27, 28, 32].

Link N peptide, a degenerated fragment of link protein of NP matrix, can support bovine NPCs survival and proliferation [32] and act as a “growth factor” to stimulate biosynthesis of proteoglycan and type II collagen [27, 33]. Moreover, link N peptide was designed to form a functionalized self-assembling peptide (RLN), and a novel self-assembling peptide nanofiber hydrogel scaffold (LN-NS) was made by mixing RLN and RADA16 peptide solution at a ratio of 1 : 1. The functionalized LN-NS can significantly enhance rabbit NPCs adhesion, differentiation, and 3D migration [16], which indicates that suitable design and self-assembly of peptide do not eliminate the bioactivity of peptide itself. Furthermore, the hydrogel property of LN-NS is able to ensure the best response of cells after cells/hydrogels mixture implanted into degenerated NP tissue [34]. Therefore, the designer functionalized LN-NS may be a suitable scaffold material for NP tissue regeneration. In addition to bioscaffold, seed cells are also a critical factor for NP tissue engineering. Many studies have showed that cells of degenerating discs are unable on their own to produce/maintain a functional ECM, which ultimately cause the loss of disc function and structure [11]. In this study, we extend our investigations to evaluate the effect of the functionalized LN-NS on rabbit BMSCs adhesion, proliferation, and differentiation* in vitro*.

Our experiments demonstrate that the functionalized LN-NS was better suited for rabbit BMSCs than the pure RADA16 hydrogel. The functionalized LN-NS exhibited excellent biocompatibility with BMSCs and significantly improved cell adhesion. As it shows in Figure 5, significant cell adhesion on the functionalized LN-NS was observed just at 1 h, which was faster than the pure RADA16 hydrogel. The fast and efficient cells-hydrogel adhesion may be attributed to the specific binds between link N peptide and BMP-R II on BMSCs [22]. Moreover, changes in cell morphology were also observed in the two peptide hydrogels, which is consistent with a recent report where cells encapsulated in RADA16 and KLD12 hydrogel have a significant change in cell morphology [20]. The presence of differing cell morphologies may be a feature of cell-cell communication. However, differing cytoskeletal morphologies were not observed in stiffer agarose hydrogel [20]. Therefore, self-assembling peptide nanofiber hydrogel may provide a more physiological microenvironment to mediate cell-cell interaction. Although the exact mechanism is not clear, these changing cell morphologies may be related to scaffold mechanical stiffness [20], cells-scaffold adhesion, and scaffold microstructure. In our study, we also evaluated the influence of the functionalized LN-NS on BMSCs proliferation. Similar cell growth was observed in the functionalized LN-NS and pure RADA16 hydrogel, which is inconsistent with the findings of Mwale et al. [32]. The conflicting results may be related to different culture time and BMSCs rapid proliferation.

Of note, our study demonstrates the potential of BMSCs chondrogenic differentiation induced by the functionalized LN-NS. In this study, we evaluated two major chondrogenic ECM genes,* coll 2* and* agg*. Consistent with previous reports on chondrogenesis of BMSCs in 3D culture scaffold, such as agarose [10], alginate [8], and KLD peptide hydrogel [20], the functionalized LN-NS and pure RADA16 hydrogel both upregulated mRNA transcript levels for* coll 2* and* agg* during 14 days of culture. However, these chondrogenic genes expression levels in cells encapsulated in the functionalized LN-NS were significantly higher than in the nonfunctional pure RADA16 hydrogel, and the cells in the functionalized hydrogel significantly increased the expression of chondrogenic genes just at 7 days. Proteins analysis further confirmed RT-PCR results. Immunofluorescence showed that BMSCs seeded on the functionalized LN-NS exhibited a higher biosynthesis level of type II collagen in comparison to the pure RADA16 hydrogel. These exciting results may be associated with the bioactive link N motif incorporated in the functionalized LN-NS. A recent study found that link N peptide can upregulate the expression of BMP-4 and BMP-7 in cells to promote biosynthesis of type II collagen and aggrecan [22].

In addition to good biocompatibility and biofunctionality, these designer functionalized self-assembling peptide scaffolds have 3D nanofiber networks, ~35 nm in fiber diameter with pores between 5 and 200 nm, and contain over 99 wt% water content [12, 14–16, 21, 26], which favors free diffusion of nutrients, bioactive factors, oxygen, and metabolites. These characteristics may be important for avascular NP tissue regeneration. Moreover, self-assembling peptide hydrogels can be injected into the NP tissue and work as a system for growth factors delivery [35, 36] and control their release. In this system, synergistic effect between functionalized hydrogel and growth factors might significantly promote degenerating NPs tissue regeneration.

## 5. Conclusions 

In this study, we extend our investigations to evaluate the effect of the designer functionalized LN-NS on BMSCs. Our findings showed that the functionalized LN-NS was nontoxic and could significantly improve BMSCs adhesion and induce BMSCs chondrogenic differentiation. Although the functionalized LN-NS has excellent biocompatibility and bioactivity with BMSCs* in vitro*, whether the LN-NS and BMSCs have the same roles* in vivo* is also unknown, which need to be further studied to determine the clinical feasibility of the novel functionalized nanofiber scaffold.
